# A Giant Ureteral Stone without Underlying Anatomic or Metabolic Abnormalities: A Case Report

**DOI:** 10.1155/2013/236286

**Published:** 2013-11-13

**Authors:** Selcuk Sarikaya, Berkan Resorlu, Ekrem Ozyuvali, Omer Faruk Bozkurt, Ural Oguz, Ali Unsal

**Affiliations:** Department of Urology, Kecioren Training and Research Hospital, Kecioren, 06380 Ankara, Turkey

## Abstract

A 28-year old man presented with left flank pain and dysuria. Plain abdominal film and computed tomography showed a left giant ureteral stone measuring 11.5 cm causing ureteral obstruction and other stones 2.5 cm in size in the lower pole of ipsilateral kidney and 7 mm in size in distal part of right ureter. A left ureterolithotomy was performed and then a double J stent was inserted into the ureter. The patient was discharged from the hospital 4 days postoperatively with no complications. Stone analysis was consistent with magnesium ammonium phosphate and calcium oxalate. Underlying anatomic or metabolic abnormalities were not detected. One month after surgery, right ureteral stone passed spontaneously, left renal stone moved to distal ureter, and it was removed by ureterolithotomy. Control intravenous urography and cystography demonstrated unobstructed bilateral ureter and the absence of vesicoureteral reflux.

## 1. Introduction 

Stones may be located in different anatomical locations of urinary tract; however, ureteral stones are usually located in three anatomic stenotic sites of ureter [1, 2]. Stone size, in particular the maximum diameter, is the most important factor dictating how a stone should be managed [2–4]. In general, ureteral stones larger than 10 mm in diameter are less likely to be passed and the majority of these patients require intervention [5]. Extracorporeal shock wave lithotripsy (SWL) and ureteroscopy (URS) are the main treatment modalities for these stones [6]. However some ureteral calculi show silent progression to reach a large size and can be larger than 10 cm in length or weighing more than 50 gram. These stones are called giant ureteral stones and seen extremely rare [5, 7]. In this study we report a case of giant ureteral calculi with ipsilateral renal calculi and contralateral distal ureteral calculi without underlying metabolic or anatomic abnormalities, which to our knowledge has not been reported before.

## 2. Case Report

**Figure 1:**
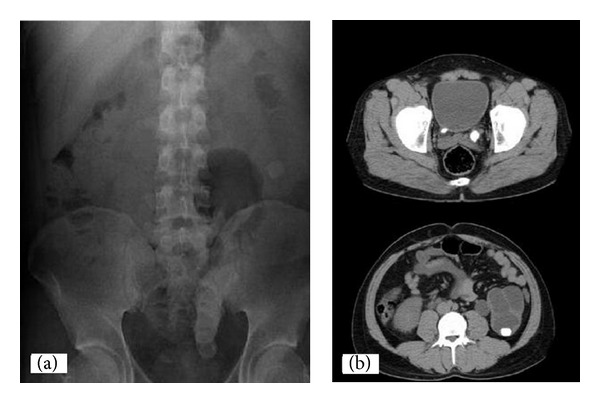
Plain film shows a long radiopaque density (11.5 cm) suggesting a giant ureteral stone in the pelvic area (a). The CT scan of the abdomen shows a left giant ureteral stone measuring 11.5 cm causing ureteral obstruction and other stones 2.5 cm in size in the lower pole of ipsilateral kidney and 7 mm in size in distal part of right ureter (b).

A 28-year-old man presented with bilateral flank pain and dysuria. Urinalysis revealed microscopic hematuria and pyuria. Urine culture was positive for *Proteus mirabilis* and was treated with ceftriaxone 1 g twice a day for 5 days. The urine culture became negative before the operation. Serum creatinine level was 1.9 mg/dL and other laboratory studies revealed no significant abnormalities. Physical examination did not yield anything apart from tenderness in left costovertebral angle location. Ultrasound (US) detected hydronephrosis and hydroureter on the left side. A plain abdominal film (KUB) and computed tomography (CT) showed a left giant ureteral stone measuring 11.5 cm causing ureteral obstruction and other stones 2.5 cm in size in the lower pole of ipsilateral kidney and 7 mm in size in distal part of right ureter (Figure 1).

**Figure 2:**
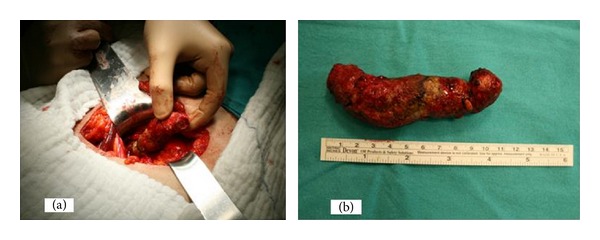
Ureterolithotomy operation (a) and removed giant ureteral stone (b).

A left ureterolithotomy operation was performed and stone was removed through a longitudinal incision (Figure 2). The ureteral wall was thick and chronically inflamed. Therefore a double J stent was inserted into the ureter and incision was closed. The stone was measured 11.5 cm in length and composed of magnesium ammonium phosphate (75%) and calcium oxalate (25%). The patient was discharged from the hospital on postoperative day 4 without any complication. The double J stent was removed under brief anesthesia 14 days postoperatively.

One month after surgery, right ureteral stone passed spontaneously. For the management of left renal stone we planned to perform percutaneous nephrolithotomy; however, on the follow-up period this stone moved to distal ureter. So it was removed by ureterolithotomy again. Control intravenous urography and cystography demonstrated unobstructed bilateral ureter and the absence of vesicoureteral reflux. A primary metabolic evaluation was performed including urine pH, serum calcium, phosphorus, uric acid, and 24-hour urinary calcium, phosphorus, oxalate, citrate, uric acid, creatinine, and electrolytes. We did not find any significant abnormalities in this evaluation.

## 3. Discussion

Stone size and location are the most important factors used to predict the likelihood of spontaneous passage in patients with ureteral stones [2–4]. The American Urological Association (AUA) guidelines, which are based on a meta-analysis of the literature, indicate that up to 98% of ureteral calculus 4 mm or smaller will pass spontaneously [8]. Furthermore frequency of spontaneous passage of stones in the mid and distal ureter was significantly higher than that of stones in the proximal ureter [9]. 

Large ureteral stones frequently cause pain and infection because of stone impaction and pelvicaliceal system obstruction [10]. This condition may result in partial or even complete loss of the renal functions if the treatment is not done promptly [11]. Currently SWL and URS are the most widely used noninvasive treatment modalities for ureteral stones. However, these minimally invasive techniques are not usable for complex large stones [6]. The management of large ureteral calculus depends on the function of the affected kidney and can require nephroureterectomy or the removal of the stones [12].

Giant ureteral stones (more than 10 cm in length or 50 gram in weight) are extremely rare in the literature. In 1992, Sabnis et al. reported the largest ureteric stone in the literature measuring 13 cm in length and weighing 90 gram [7]. However the etiology and pathology of these stones remain unclear. Some authors have reported giant ureteral stones in association with ureteral duplication, ureteroceles, tuberculosis, megaureter, or prolapsed benign polyp of the ureter [12–15]. Therefore a urinary tract abnormality or a metabolic defect may play an important role in the pathogenesis of these stones. But in our case we could not find any anatomic or metabolic abnormalities.
